# Granulomatous mastitis, erythema nodosum, and arthritis syndrome: a narrative review

**DOI:** 10.3389/fimmu.2025.1689710

**Published:** 2025-12-08

**Authors:** Sheng-Guang Li, Jing Zhang, Ji Li, Lina Zhang, Yadan Zou, Ting Long, Ruohan Yu, Yanfeng Zhang

**Affiliations:** Department of Rheumatology and Immunology, Peking University International Hospital, Beijing, China

**Keywords:** idiopathic granulomatous mastitis (IGM), erythema nodosum (EN), arthritis, GMENA syndrome, autoimmune mastitis, immunosuppressive therapy, breast disease, narrative review

## Abstract

**Introduction:**

Idiopathic granulomatous mastitis (IGM) is a rare chronic inflammatory breast disease of unknown etiology, typically presenting with breast pain, swelling, and mass formation. Erythema nodosum (EN) is an inflammatory panniculitis that can occur as a systemic manifestation of various conditions, and arthritis has occasionally been observed alongside granulomatous mastitis (GM), suggesting an underlying autoimmune component. This narrative review synthesizes reported cases of GM with EN, with or without arthritis—termed “GMENA” syndrome—to characterize its clinical features and management.

**Methods:**

We searched PubMed and EMBASE for case reports and case series describing GM with co-occurring EN, with or without arthritis. Reports fulfilling these criteria were reviewed, and data on demographics, clinical presentation, laboratory and histological findings, treatment strategies, and outcomes were extracted and summarized descriptively.

**Results:**

We identified 45 reported patients with GMENA in the literature. All patients were women of reproductive age (median age approximately 32 years), and about one-third presented during pregnancy or in the postpartum period. Breast involvement was exclusively unilateral, manifesting as painful inflammatory masses often mimicking carcinoma. EN was universal (100%), usually affecting both shins and ankles, and approximately two-thirds of patients developed acute, non-erosive oligoarthritis, most commonly involving the ankles. Laboratory work-ups consistently showed elevated inflammatory markers with negative autoantibodies and sterile cultures. Histology typically revealed lobulocentric, non-caseating granulomas with neutrophilic microabscesses. Corticosteroids induced remission in more than 75% of cases, while methotrexate or azathioprine provided steroid-sparing benefit in refractory disease. Antibiotics and anti-tuberculous regimens were generally ineffective, and surgery was mainly used for biopsy, drainage, or limited excision. Relapses (about 13%) were uncommon and often followed rapid tapering of corticosteroids.

**Discussion:**

GMENA appears to represent a distinct clinicopathologic entity within the spectrum of IGM, characterized by the triad of granulomatous mastitis, EN, and frequently acute oligoarthritis in women of childbearing age. The available evidence supports an underlying immune-mediated mechanism and favors systemic corticosteroids, with early introduction of steroid-sparing immunosuppressants in refractory or relapsing disease. Antibiotics and extensive surgery have limited roles. Further prospective and mechanistic studies are needed to clarify pathogenesis and to optimize long-term management strategies.

## Introduction

Idiopathic granulomatous mastitis (IGM) is an uncommon, benign inflammatory breast disease defined by noncaseating, lobulocentric granulomas. First reported by Kessler and Wolloch in 1972 (1), it most often affects young parous women, frequently within a few years of pregnancy or breastfeeding (2). Clinically, IGM presents as a unilateral painful mass with overlying erythema or induration that may mimic abscess or carcinoma. Because its etiology remains uncertain, IGM is a diagnosis of exclusion after ruling out infectious granulomatous mastitis (e.g., *Mycobacterium tuberculosis* or fungi) and systemic granulomatous disorders such as sarcoidosis.

Several factors have been proposed to contribute to disease onset. Hormonal/reproductive influences: The predominance in women of childbearing age and frequent postpartum/lactational onset suggest a possible autoimmune reaction to extravasated milk antigens within ducts (3). Hyperprolactinemia and oral contraceptive use have also been postulated as triggers (4). Infectious hypotheses: Corynebacteria have been detected in a subset of lesions (5), with some studies isolating *Corynebacterium* species in 41% of IGM cases (5, 6); nevertheless, no single pathogen has been proven causal, and many cases are culture-negative. Immune-mediated mechanisms: Failure to identify infection, the granulomatous histology, and the prompt response to corticosteroids collectively support an immune basis (7, 8), and co-occurring immune phenomena such as erythema nodosum (EN) and arthritis further imply an autoinflammatory/autoimmune process (2).

EN is an inflammatory panniculitis of subcutaneous fat that typically manifests as tender red nodules on the extensor legs and is widely regarded as a hypersensitivity reaction to systemic antigenic stimuli (e.g., streptococcal infection, medications, sarcoidosis, inflammatory bowel disease) (9–11). Although EN can be idiopathic, it often signals an underlying systemic process.

Against this background, a small but growing number of patients with IGM have been reported to develop EN concurrently. The first documented coexistence was described by Adams et al. in 1987 (12), in whom granulomatous mastitis occurred with EN and polyarthritis. Since then, scattered reports have continued to describe this triad of granulomatous mastitis, erythema nodosum, and arthritis—now often referred to as the GM–EN (± arthritis) combination—although it remains rare.

**Figure 1:**
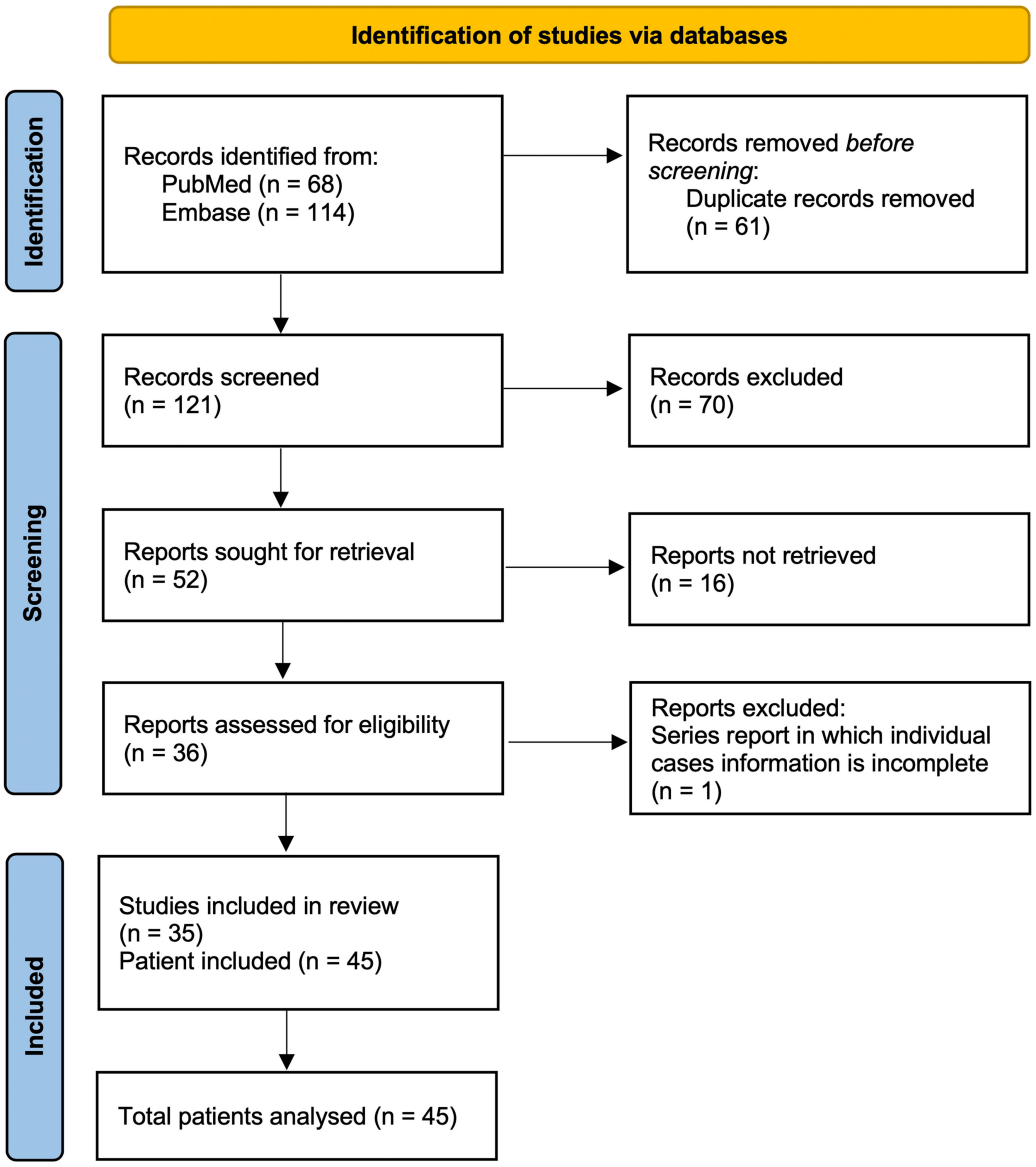
Literature selection summary of the reviewed publications on GMENA syndrome. We systematically searched PubMed (*n*=68) and Embase (*n*=114) through Augst 2025 using combinations of “idiopathic/granulomatous mastitis” with “erythema nodosum/arthritis.” After removing duplicates (*n*=61), 121 records were screened; 70 were excluded on title/abstract. Full texts were sought for 52 reports; 16 were not retrieved. A total of 36 reports were assessed in full; one series was excluded because individual case information was incomplete. In total, 35 studies were included, comprising 45 patients analyzed in this review. Inclusion required idiopathic granulomatous mastitis with EN (± arthritis) and extractable individual data; exclusions included infectious granulomatous mastitis (e.g., TB/fungal) or reports without EN. *Abbreviations:* GM, granulomatous mastitis; EN, erythema nodosum; TB, tuberculosis.

By 2015, fewer than a dozen cases had been published. With increasing recognition, a 2021 review identified 29 patients across 14 reports (13), and additional cases published thereafter bring the aggregate to 45 as of 2025 (see **Figure 1** for our literature search strategy).

This review synthesizes the available evidence on granulomatous mastitis associated with EN, emphasizing clinical presentation, immunological features, controversies in pathogenesis, and management. By analyzing all reported cases, we aim to delineate the characteristic features of this underrecognized syndrome, consider whether it represents a distinct autoimmune entity, and highlight knowledge gaps and priorities for future research.

## Literature review methodology

For this narrative review, we performed a comprehensive literature search to identify reported cases of granulomatous mastitis occurring with erythema nodosum, with or without associated arthritis. Searches of the PubMed and Embase databases (last conducted in August 2025) were undertaken using keywords related to “granulomatous mastitis,” “erythema nodosum,” and “arthritis.” Relevant articles were identified through title and abstract screening, and their references were reviewed to ensure that all pertinent reports were included. Given the rarity of GMENA, all case report and case series publications meeting the abovementioned criteria were included without methodological quality restrictions. **Figure 1** provides a summary of the literature selection process. Key data from the published cases were synthesized qualitatively and tabulated (**Tables 1** and **2**) to facilitate a descriptive analysis.

**Table 1:** Clinical characteristics of granulomatous mastitis with erythema nodosum ± arthritis (45 reported cases).

Characteristic	Data (*N*=45)
Demographics	All female (100%); age range 16–43, median ~32 years
Pregnancy/postpartum	16 cases (35.6%) postpartum or pregnant; 29 (64.4%) non-puerperal
Breast lesion laterality	Unilateral 45 cases (22 right, 23 left); bilateral: 0 cases
Breast clinical features	Painful hard breast mass in 100%. Skin erythema in 100%. Peau d’orange or nipple retraction in ~20%. Axillary nodes in ~20%. Sinus/abscess formation in ~30%
Histopathology (breast)	Non-caseating granulomatous lobulitis with neutrophils in 100%. Giant cells common. Cultures negative in all idiopathic cases (1 *Corynebacterium* isolate; 1 AFB stain+ in 1987 case)
Erythema nodosum (EN)	All 45 had EN. Distribution: Both legs in 40 (88.9%)—mainly shins/ankles; +forearms in 3 (6.7%); unilateral leg in 1. Plantar foot in 1 Lesions often multiple (≥5 nodules) and tender. EN onset concurrent with or shortly after mastitis in most
Arthritis	30 cases (66.7%) had arthritis/arthralgia; 15 (33.3%) had no significant joint symptoms. Most involve both ankles, knees, wrists, elbows, and hands. Typically acute, non-erosive oligoarthritis
Fever	Present in 29 (42.2%)—usually low-grade (<39 °C). Absent in 26 (57.8%)
Laboratory	Elevated ESR/CRP in 64.4%. Leukocytosis in ~50%. Mild anemia ~30%. Autoimmune serologies—ANA, RF, etc.—negative in all tested (except positive aPL in 1). ACE levels normal in all tested (no sarcoid). TB/fungal tests/cultures all negative (idiopathic GM by exclusion)
Sarcoidosis evidence	None—chest imaging normal in 100%. No uveitis or other organ involvement reported

**Table 2:** Treatments and outcomes in granulomatous mastitis with erythema nodosum ± arthritis (*N*=45).

Treatment/Outcome	Frequency in cases (*N*=45)
Antibiotics or anti-TB given	9 cases (20.0%)—empirical therapy prior to diagnosis. Little/no improvement in mostscirp.org
Corticosteroids used	40 cases (88.9%)—nearly all patients ultimately received prednisone (typically 30–60 mg/day) or equivalent. Steroid therapy led to a marked improvement in the majority
NSAIDs used	20 cases (44.4%)—for adjunct pain/inflammation control (EN, arthritis). Provided symptom relief; not sufficient alone
Immunosuppressants (overall)	8 cases (17.8%)—used adjunctively for steroid-sparing or refractory disease
– Methotrexate	6 cases—most common steroid-sparing agentskin.dermsquared.com. Effective in maintaining remission or inducing remission when steroids inadequate
– Azathioprine	2 cases—used in place of or with MTX (some reports). Benefit noted
Surgical intervention	9 cases (20%)—including 5 with core/excisional biopsy or abscess drainage and 4 with wider excision/segmental resection. Surgery alone did not cure the disease but helped in diagnosis or debulking. No mastectomy performed in any case reviewed
Clinical outcome—remission	34 cases (75.6%) achieved complete remission of breast and EN lesions with treatment and remained disease-free at the last follow-up
Relapse of GMENA	6 cases (13.3%) had a relapse after initial improvement. Triggers included tapering off steroids too quickly or a subsequent pregnancy. All relapses were successfully re-treated (no case was chronically active after re-treatment).
Partial remission	3 cases (~6.7%) had partial response: EN resolved but granulomatous mastitis persisted as a milder chronic lump (or vice versa). These cases are often managed with longer immunosuppression; no progression noted
Lost to follow-up	1 case (2.2%)—outcome not reported
Mortality or malignancy	0 cases—no deaths attributable to GMENA. No transformation to malignancy (IGM is benign; no case of breast cancer was reported in these patients)

### Synthesis of clinical reports

Literature search and selection: We identified all published cases of IGM with EN (± arthritis) meeting the abovementioned criteria. The 45 patients (from 35 studies) were all female and generally of reproductive age. Geographically, cases have been reported across Europe, Asia, the Middle East, and the Americas. The aggregated clinical features are summarized in **Table 1**. Notably, EN was present in 100% of cases by inclusion criteria, and approximately two-thirds of the reported cases feature arthritis/arthralgias in addition to EN (2, 3, 8, 12–31), whereas about one-third do not (20, 32–44). No included patient had evidence of an alternative granulomatous disease (e.g., sarcoidosis) or an infectious cause. In the section below, we describe the clinical characteristics, immunological findings, management, and outcomes observed in these patients.

Patient demographics: All reported cases have occurred in women. Ages ranged from 16 to 43 years, with a typical age in the 30s, consistent with idiopathic granulomatous mastitis. Many—but not all—patients had a recent obstetric history. In the 29 cases summarized by Parperis et al. (2021), 20.7% developed GM during pregnancy (13); several presented in the third trimester, suggesting that gestational immune-hormonal shifts may precipitate disease. Conversely, GM–EN has also occurred in nulliparous patients or without recent pregnancy. Cases have been described across diverse ethnic and geographic backgrounds (Europe, Asia, Middle East, Americas); no clear racial predisposition is evident, although one Spanish cohort included many patients of Latin American origin (45).

Breast involvement: In GMENA, breast involvement is predominantly unilateral, but rare bilateral cases have been reported; these bilateral cases often correspond to a more refractory disease (46). In a comparative cohort, 2/43 EN-positive IGM cases were bilateral versus 0/43 EN-negative controls (47). Local signs usually include a painful enlarging mass with overlying erythema; nipple retraction/dimpling and sinus tract/ulceration can occur. Compared with isolated IGM, EN-associated cases show more active disease, with larger masses, higher inflammatory markers (WBC/ESR/CRP), and higher rates of fistula and recurrence, supporting a more exuberant inflammatory phenotype. Constitutional symptoms vary; some patients report malaise or weight loss, and a subset have fever (2, 31, 44).

Erythema nodosum (EN): By definition, all patients in this series developed EN, usually concurrent with or shortly after the breast manifestations. Lesions are multiple, tender, erythematous nodules—most often on the anterior shins; ankles, thighs, or forearms may also be involved (3, 19). Clinicopathology matches idiopathic EN (septal panniculitis without vasculitis), and lesions typically resolve without scarring. Importantly, EN can expedite diagnosis: in one study, patients with EN were diagnosed much earlier on average (~0.4 months from onset) than those without EN (~6 to 7 months) (45).

Arthritis: Approximately two-thirds of reported cases (≈66.7%) experienced peripheral arthritis or arthralgias in addition to EN (2, 3, 8, 12–31), while about one-third did not (20, 32–44). In the 2021 review by Parperis et al., nine of 29 cases (31%) had arthritis (13). When present, the joint involvement is typically an acute, non-erosive oligoarthritis of peripheral joints—most often affecting both ankles (frequently bilateral) but also knees and wrists (7, 8, 14, 18, 27, 28, 30). The combination of EN with bilateral ankle arthritis recalls Löfgren’s syndrome in sarcoidosis and has fueled debate about a possible sarcoid-related spectrum (3, 48). Joint pain and stiffness may limit mobility (including during pregnancy), yet symptoms usually improve in tandem with EN and breast inflammation under appropriate therapy (23).

Laboratory findings: Testing shows a general inflammatory profile—elevated ESR/CRP in ~64% of cases and mild leukocytosis in ~50%—but lacks disease-defining autoantibodies (ANA, RF, anti-dsDNA, ANCA, etc., all negative). Other abnormalities (e.g., mild anemia in ~30%) are inconsistent and often incidental. Crucially, infectious evaluations are negative: cultures/PCR from breast tissue or abscess fluid do not yield pathogens, and *Mycobacterium tuberculosis* is excluded by either negative microbiology or pathology inconsistent with TB (no caseation, no acid-fast bacilli) (23). Serologic tests for common EN triggers (e.g., streptococcal infection, IBD) are usually unrevealing.

**Figure 2:**
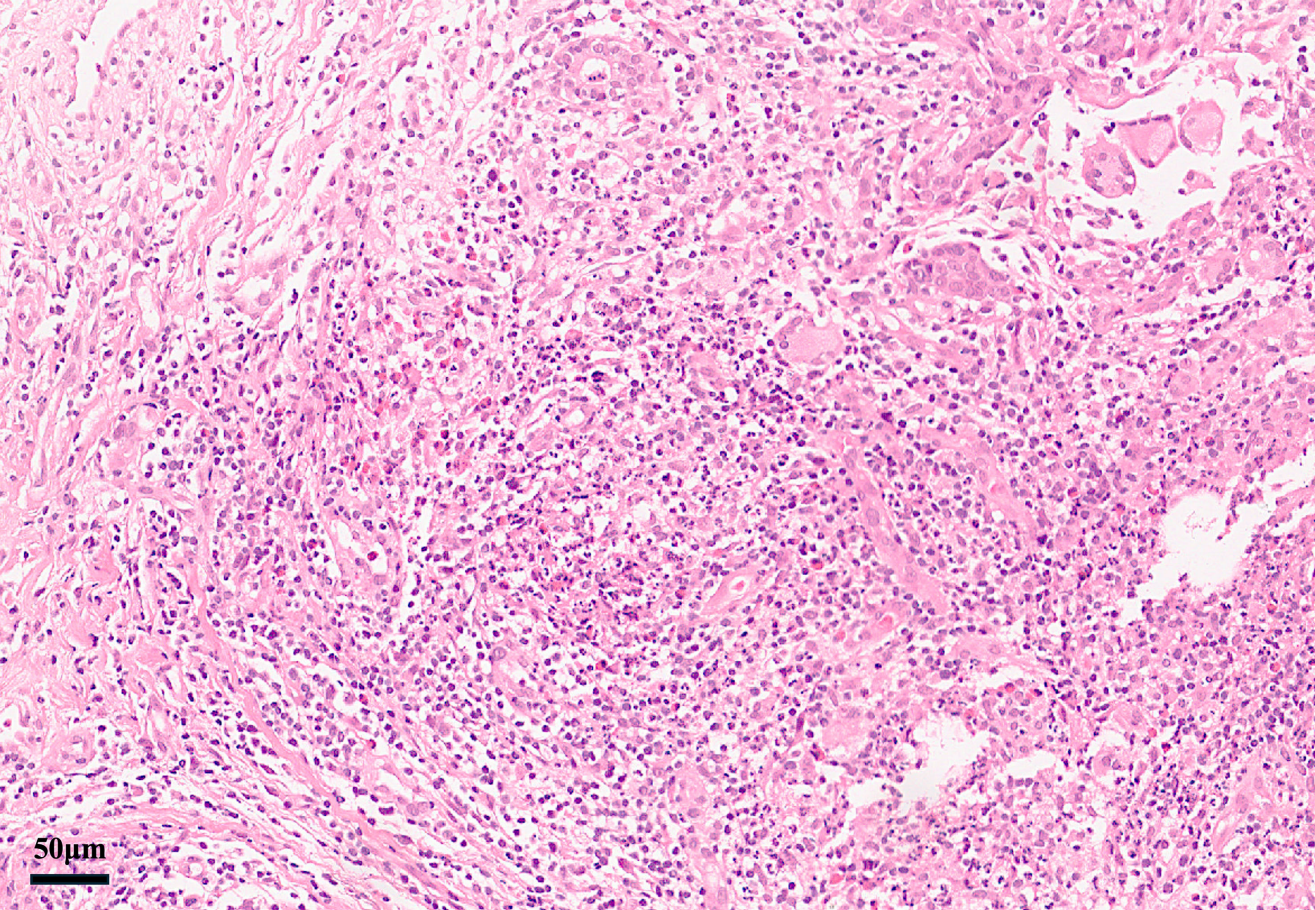
Breast core biopsy in GMENA showing granulomatous lobular mastitis (H&E). Lobulocentric noncaseating granulomas with mixed inflammatory infiltrate and focal neutrophilic microabscesses are visible. No caseous necrosis is present. These features support idiopathic granulomatous mastitis. Reproduced with permission from the original author (44).

Pathology: Diagnosis requires breast tissue biopsy demonstrating granulomatous lobular mastitis with noncaseating granulomas centered on lobules, often accompanied by neutrophilic microabscesses and multinucleated giant cells (49). Special stains for organisms (Gram, Ziehl–Neelsen, fungal) are negative, and no malignant cells are present (50). When biopsied, EN lesions show a septal panniculitis without vasculitis, fully consistent with typical EN (51). Granulomas are not seen in EN skin, which helps distinguish these cases from sarcoidosis-specific skin lesions (3). **Figure 2** illustrates a representative breast pathology from the reported cases.

Treatment and outcomes: **Table 2** summarizes therapies and outcomes in the 45 cases. Management generally targeted the underlying immune-mediated inflammation, namely:

- Glucocorticoids: Systemic corticosteroids were the first-line therapy in the vast majority of cases (89% received prednisone or equivalent). Doses were typically 0.5–1 mg/kg/day (e.g., 40–60 mg prednisone), leading to rapid improvements in breast and skin lesions within 1–2 weeks (31, 41, 47). Parperis et al. noted an 86% symptom improvement rate with steroids (13). Tapering was gradual over months. Relapses often occurred with overly rapid taper or postpartum (52), but all relapses responded to re-initiation of steroids or addition of immunosuppressants. Some authors kept patients on low-dose prednisone for ≥6 months to ensure full remission (53).
- Antibiotics and anti-tuberculous therapy: Empiric antibiotics were often tried initially (prior to diagnosis) but were ineffective once idiopathic GMENA was established. In 20% of cases, the patients received prolonged antibiotics for presumed infection with little or no improvement. Similarly, a few cases received anti-TB therapy empirically, but in confirmed idiopathic GM (with negative TB tests), there was no benefit. These observations support a non-infectious pathogenesis.
- Steroid-sparing agents: To minimize steroid exposure or treat a refractory disease, immunosuppressive agents were used in ~18% of cases. Methotrexate (MTX, 15–25 mg weekly) was the most common adjunct and was used in six cases (3, 7, 20, 25, 26, 29), often enabling steroid taper and maintaining remission. Azathioprine or mycophenolate mofetil were used in a few cases (each in one to two patients) with reported benefit (14, 18, 26).
- NSAIDs and others: Non-steroidal anti-inflammatory drugs were frequently given for adjunct pain and inflammation control (especially for EN or arthritis symptoms), although they did not suffice as sole therapy (54, 55). Dapsone has been reported in one case of milder disease, with some benefit for skin lesions. Colchicine or hydroxychloroquine (therapies classically used for EN) were not commonly utilized in these reports (21, 56).
- Biologic therapy: Biologics (e.g., TNF-α inhibitors) were rarely reported but are a theoretical option in refractory cases. At least one IGM case (without EN) responded to adalimumab after failure of conventional therapy (57). In our review, no GMENA case was treated with biologics as of 2025. The use of biologics should be reserved until infection (especially TB) is rigorously excluded.
- Surgery: Surgery is generally considered a second-line or adjunctive measure. Only a minority of patients (20%) required any surgical procedures, most commonly core-needle biopsy, abscess drainage, or limited excisional biopsy for diagnosis or relief. Parperis et al. reported that ~14% of cases underwent some surgical intervention (13). Extensive surgery (wide excision or mastectomy) is not a first-line approach due to significant morbidity and because it does not address the systemic features (EN, arthritis). Operations have typically been reserved for complications (e.g., persistent abscess/fistula) or for diagnostic purposes, and some residual masses were electively excised after inflammation subsided for symptom or cosmetic reasons. However, Çetin et al. in 2020 observed that more than half of EN-associated IGM patients failed steroid therapy and ultimately required surgical excision (including mastectomy) (46). While surgery is not a first-line strategy for GMENA, when an operation is pursued for refractory disease or persistent fistula, current consensus documents recommend planning for wide local excision rather than limited excision to minimize recurrence (often reported as low as ~5% when performed after inflammatory quiescence) (58, 59).
- Outcomes: Prognosis was generally favorable with appropriate therapy. Complete remission of breast and skin lesions was achieved in ~75.6% of cases, with follow-up periods of 6–24 months in many reports. Only six cases (13.3%) experienced a relapse after initial remission (2, 8, 25, 30, 31, 39); all relapses were successfully re-treated with steroids (often with an immunosuppressive added). Partial remission (persistence of a mild breast lesion or residual EN) was noted in ~6.7%, often managed with longer courses of therapy (17, 21, 22). No case progressed to systemic sarcoidosis or another autoimmune disease during available follow-up, and no malignancies or deaths were attributed to GMENA.

## Discussion

Underrecognized but clinically important: GMENA syndrome remains underrecognized. Because IGM itself is uncommon, many patients initially receive repeated antibiotics for presumed infection before the correct diagnosis is made. Awareness that EN in a patient with mastitis is a diagnostic clue can shorten delays: early breast biopsy and rheumatology/dermatology input are advised. Conversely, in a woman with unexplained EN ± acute ankle arthritis, directed questioning and a breast examination may uncover occult mastitis. Naming the entity (e.g., using the proposed term “GMENA syndrome”) may aid recognition and education.

### Differential diagnosis and diagnostic workup

In patients presenting with an inflammatory breast mass and concomitant erythema nodosum (EN), a high index of suspicion and multidisciplinary assessment (breast surgery/radiology, dermatology, rheumatology) are essential. Tissue confirmation is mandatory. Begin with ultrasound ± mammography and drain if a fluctuant abscess is suspected; when mastitis is unexplained or persistent—especially in the presence of EN—obtain a core-needle biopsy to distinguish idiopathic granulomatous mastitis (IGM) from carcinoma, tuberculosis, or other granulomatous diseases (14, 29, 49). Histology in IGM shows lobulocentric, non-caseating granulomas often with neutrophilic microabscesses, whereas infectious causes favor caseation or organism identification; therefore, send AFB and fungal stains/cultures and consider PCR to exclude TB and deep mycoses (14, 22, 49). Because EN frequently reflects a systemic trigger, perform a standard EN workup (e.g., chest imaging for sarcoidosis, streptococcal testing, interferon-γ release assay/PPD for TB, targeted stool/IBD evaluation when indicated, and basic autoimmune serologies) (29). In reported GM-EN/GMENA cases, these ancillary studies are typically unrevealing, supporting a non-infectious, immune-mediated process once malignancy and infection are excluded.

### Key differentials to exclude

(1) Inflammatory breast carcinoma can mimic mastitis (erythema/skin thickening) (60, 61), but biopsy reveals malignancy rather than granulomas; this is the principal rule-out when imaging is worrisome (2). Infectious mastitis, particularly tuberculous mastitis in endemic settings, favors caseating granulomas and/or positive stains/cultures or PCR; fungal or other atypical infections should be considered in non-responders and sampled appropriately (14, 22, 62) (3). Sarcoidosis may present with EN and ankle arthritis (Löfgren’s syndrome) (63, 64), and sarcoid of the breast is described; findings such as bilateral hilar lymphadenopathy, granulomas in other organs, or elevated ACE support sarcoidosis over organ-limited IGM/GMENA (4). Other autoimmune conditions rarely involve the breast but enter the differential—e.g., granulomatosis with polyangiitis (necrotizing vasculitis on pathology) (65) or rheumatoid nodules (66)—and should be screened clinically as appropriate. Finally, coincidence of idiopathic EN with unrelated puerperal mastitis is possible; however, the recurrent co-occurrence of GM with EN and the parallel response to immunosuppression across reports argue for a linked immune phenomenon rather than chance (2, 13).

### Pathogenesis

The pathogenesis of GMENA syndrome remains speculative. It likely involves a complex interplay of immune dysregulation and possibly genetic predisposition. Some authors have postulated that the granulomatous inflammation in the breast could expose or release antigens that trigger a systemic immune reaction, manifesting as EN and arthritis (13). Others have suggested that an underlying predisposition (such as an inherent tendency for granulomatous inflammation) might be unmasked by an initial event like pregnancy-related breast changes or a minor infection. The observation that a significant subset of cases occurred in the peripartum period raises questions about hormonal or lactation-related triggers. Prolactin levels and breast engorgement might play a role in initiating granulomatous mastitis, which then provokes a systemic immune response—a hypothesis supported by the “prolactin theory” in IGM (67) and studies exploring PRL signaling in immune regulation. Single-cell transcriptomic data further show that in granulomatous mastitis tissues, the mammary epithelium exhibits upregulation of prolactin downstream pathways (JAK/STAT, MAPK) and shifts in immune cell phenotype (e.g., pro-inflammatory macrophage subsets, activated IFN-γ and NF-κB signaling) (68). Some case reports have also documented alterations in T cell, NK, and NKT cell counts in IGM patients, consistent with systemic immune dysregulation (69). Finally, broad immunologic reviews and marker studies in IGM emphasize that cytokine imbalances (IL-2, IL-6, TNF, etc.) are frequently observed in affected patients (70, 71). However, current evidence is limited to case data and molecular explorations, and more focused mechanistic research is needed to clarify these pathways.

### Autoimmune disease vs. limited sarcoidosis

Whether GMENA is a distinct autoimmune/autoinflammatory syndrome or falls within the sarcoidosis spectrum remains uncertain. Some authors have interpreted the triad (GM with EN ± bilateral ankle arthritis) as an acute sarcoid-like presentation—even when chest imaging is unremarkable—based on its resemblance to Löfgren-type patterns (3). Others emphasize the organ-limited nature (breast ± EN), sterile microbiology, and excellent response to immunomodulation, arguing for an organ-specific immune process rather than systemic sarcoidosis (13, 72, 73). Given the case-based evidence and absence of definitive immunologic markers, both interpretations are plausible, and a chance co-occurrence of idiopathic EN with GM cannot be excluded. Accordingly, we adopt neutral terminology and frame management pragmatically (infection exclusion, immunomodulation first, surgery reserved for complications).

### Role of infectious agents

Whether bacteria—particularly *Corynebacterium*—act as drivers or bystanders remains uncertain. In at least one EN-associated case, *Corynebacterium* was isolated, yet the patient improved only with corticosteroids, arguing against infection alone (16). The detection of corynebacterial DNA in ~41% of IGM lesions (by one study) suggests a possible trigger in some cases, analogous to EN after streptococcal infection (55). Hida et al. (2014) reported a GM–EN case they considered “probably due to *Corynebacterium* infection” based on cultures (16), but no single pathogen appears consistently in GMENA reports. Modern broad-range sequencing may detect occult microbes; if a consistent organism emerges, adjunct antimicrobial therapy could be rational. At present, however, clinical experience supports a sterile, immune-mediated inflammatory process rather than an active infection.

Optimal treatment strategies: Randomized trials are lacking; management is guided by case series and related conditions. Corticosteroids reliably induce remission; the key practical issue is steroid sparing. Early addition of methotrexate in extensive or relapsing disease can facilitate tapering and may reduce relapse risk (7, 74, 75). For selected patients, therapies targeting EN or arthritis (e.g., colchicine, potassium iodide, dapsone) have been reported anecdotally (21, 56). Biologic agents (like anti-TNF) are a logical consideration in refractory cases, but strict exclusion of infection (especially TB) is mandatory before use. Questions remain about managing postpartum cases—e.g., whether to suppress lactation to prevent antigenic stimulation (evidence is limited to anecdotal reports). Developing consensus treatment algorithms (potentially through multi-center collaboration) would help guide therapy in the absence of trials.

### Research gaps

Mechanistic immunology in GMENA is largely unexplored. Key questions include the following: What T-cell subsets and cytokine profiles dominate in breast granulomas (e.g., a Th1 pattern)? Are B-cells or autoantibodies involved? Do genetic predispositions (such as HLA-B8/DR3, which is associated with Löfgren’s syndrome) play a role? Long-term outcomes are also not well defined; it is unknown whether a subset of GMENA patients later develop systemic autoimmune diseases or sarcoidosis. Establishing registries and banking tissue samples for future analyses would facilitate answering these questions.

### Potential developments

As pathogenesis is clarified, targeted immunomodulation (e.g., JAK inhibitors or IL-1/IL-23 pathway inhibitors) could be tested in refractory cases. Standardized diagnostic criteria and a multi-center case registry would improve case ascertainment and help evaluate therapies—for example, existing criteria for sarcoid arthritis (Visser et al., 2002) illustrate how structured definitions facilitate research (76). Analogous criteria for GMENA—such as biopsy-proven granulomatous mastitis plus EN, after excluding infection—could be proposed to unify case identification in future studies.

### Limitations

This narrative review is inherently limited by the nature of the available data. All information comes from isolated case reports and small series, which are subject to publication bias and often lack comprehensive documentation. The total number of reported cases (28) remains small, and there may be additional unpublished cases or cases that were not recognized as GMENA. Without controlled studies, it is not possible to determine the incidence of GMENA among all GM cases, nor can we establish definitive causality or risk factors. Nonetheless, by aggregating these reports, we gain valuable insights into common features and effective treatments, which can inform clinical practice.

## Conclusion

GMENA is an uncommon but distinctive syndrome bridging organ-specific and systemic inflammation. Current evidence suggests an immune-mediated pathogenesis (with excellent response to immunosuppression). Regardless of whether it ultimately represents a limited form of sarcoidosis or a separate autoimmune phenomenon, exclusion of infection and prompt corticosteroid therapy are crucial for good outcomes. Heightened clinical awareness—linking EN (± ankle arthritis) with granulomatous mastitis—can expedite diagnosis and improve outcomes, while ongoing immunologic studies will refine classification and guide targeted treatment.

## Data Availability

The original contributions presented in the study are included in the article/supplementary material. Further inquiries can be directed to the corresponding author.
